# Mechanistic insights into PFAS-induced effects on B lymphocyte activation and antibody secretion

**DOI:** 10.1007/s00204-026-04377-0

**Published:** 2026-04-23

**Authors:** Martina Iulini, Karsten Beekmann, Ron L. A. P. Hoogenboom, Valentina Galbiati, Giulia Russo, Francesco Pappalardo, Styliani Fragki, Alicia Paini, Emanuela Corsini, Aafke W. F. Janssen

**Affiliations:** 1 Laboratory of Toxicology, Department of Pharmacological and Biomolecular Sciences “Rodolfo Paoletti”, Università Degli Studi Di Milano, Milan, Italy; 2 Wageningen Food Safety Research (WFSR), Wageningen University & Research, Akkermaalsbos 2, 6708 WB Wageningen, The Netherlands; 3 Department of Health and Drug Sciences, Università Degli Studi Di Catania, Catania, Italy; 4 esqLABS GmbH, 26683 Saterland, Germany

**Keywords:** Antibody production, Immunosuppression, NAMs, PFASs, RNAseq, Glucocorticoid receptor

## Abstract

Per- and polyfluoroalkyl substances (PFASs) are man-made organofluoride chemicals widely present in the environment, with exposure associated to various adverse health effects, including immunotoxicity. Recently, we showed that PFASs can directly impair antibody production, leading to decreased immunoglobulin (Ig) M and IgG release in human peripheral blood mononuclear cells (PBMCs) obtained from both male and female healthy donors. However, the underlying molecular mechanisms remain largely unknown. In this study, we aimed to address this gap by performing RNA sequencing to identify pathways and genes potentially involved in the observed immunotoxic effects. PBMCs were exposed to selected PFASs for 24 h, and to assess effects on antibody secretion, a subset was subsequently stimulated with CpG oligodeoxynucleotide ODN2006 and rhIL-2 for an additional six days. Transcriptomic analysis indicated activation of the glucocorticoid receptor (GR) and associated signaling pathways, supported by the upregulation of several GR-target genes and the prediction of glucocorticoids or the GR agonist dexamethasone as upstream regulators in Ingenuity Pathway Analysis. Moreover, the inhibitory effects of PFASs on antibody secretion were shown to be reversable by the GR antagonist Mifepristone, supporting the involvement of the GR in PFAS-mediated suppression of antibody secretion. Overall, this research advances our understanding of PFAS-induced immunotoxicity and identifies potential biomarkers for evaluating PFAS exposure and its associated health effects.

**Graphical abstract:**

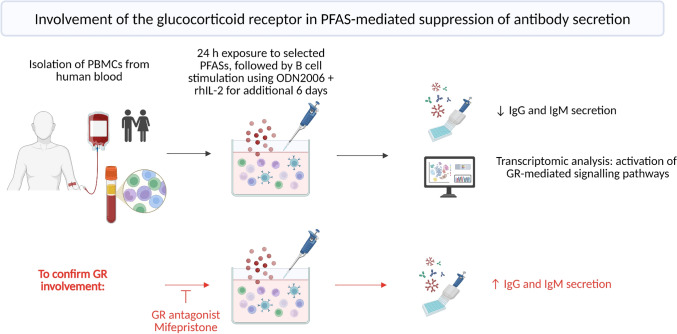

**Supplementary Information:**

The online version contains supplementary material available at 10.1007/s00204-026-04377-0.

## Introduction

Per- and polyfluoroalkyl substances (PFASs) are synthetic organofluoride compounds which have been used in a wide variety of home and industrial applications due to their unparalleled properties, among others to repel water- and grease, having a low coefficient of friction, and being resistant to heat. These properties, however, also make part of them extremely persistent in the environment and biological systems, which has earned them the nickname “forever chemicals” (Allen 2018). This environmental and biological persistence has resulted in the detection of PFASs in soil, water, wildlife, and human tissues, including blood, breast milk, and umbilical cord samples (Antoniou et al. 2022). Their ubiquitous presence has raised significant concerns for human and environmental health, and several adverse health effects are associated with PFASs exposure (Habib et al. 2024), of which immunotoxicity is considered the most sensitive endpoint at least for a number of the most ubiquitously present PFASs. The immunotoxic effects are supported by epidemiological studies (Grandjean et al. 2012; Granum et al. 2013; Abraham et al. 2020), in vivo animal experiments (DeWitt et al. 2008; Peden-Adams et al. 2008), and in vitro research (Janssen et al. 2023; Corsini et al. 2024). The European Food Safety Authority (EFSA) has identified the reduced vaccination efficacy associated with PFASs serum levels as the critical effect for risk assessment, which led to the setting of a group tolerable weekly intake for the sum of perfluorooctane sulfonate (PFOS) perfluorooctanoic acid (PFOA), perfluorononanoic acid (PFNA), and perfluorohexane sulfonate (PFHxS) of 4.4 ng/kg body weight (EFSA CONTAM PANEL et al. 2020). Similarly, the U.S. Environmental Protection Agency recognized PFASs-induced immunotoxicity as a critical effect of concern (EPA 2022), emphasizing its impact on vulnerable populations, particularly children, in its risk assessment framework.

The immune system, which is a complex network of cells and molecules responsible for defending the body against pathogens and for maintaining tissue homeostasis, appears to be particularly vulnerable to PFASs. Studies have identified various adverse effects of PFASs on immune function, ranging from reduced antibody production to impaired immune cell activity and cytokine dysregulation (Kielsen et al. 2016; Dalsager et al. 2016). Epidemiological studies have found an inverse correlation between PFOA and PFOS serum levels and vaccine-induced antibody responses, particularly for tetanus and diphtheria (Grandjean et al. 2012, 2017; Granum et al. 2013). Prenatal and early-life exposure has been linked to lower antibody levels in children (Abraham et al. 2020; Shih et al. 2021; Padula et al. 2025). Key studies have shown that maternal PFOS and PFOA serum concentrations are associated with reduced antibody levels in children, highlighting the impact of PFAS exposure on immune function (Abraham et al. 2020; Shih et al. 2021). Animal studies support evidence that PFAS exposure leads to dose-dependent reductions in serum levels of immunoglobulin M (IgM) and G (IgG), particularly affecting T cell-dependent antibody responses (TDAR) against injected antigens such as sheep red blood cells. This conclusion is supported by multiple experimental studies showing such decreases for compounds such as PFOA (Dewitt et al. 2008, 2016; De Guise & Levin 2021), PFOS (Peden-Adams et al. 2008; Dong et al. 2009; Zheng et al. 2009), hexafluoropropylene oxide dimer acid (HFPO-TA)(Rushing et al. 2017), and aqueous film-forming foam mixtures containing C5-C10 PFASs, including chlorinated PFOS (McDonough et al. 2020). Importantly, also T cell-independent antibody responses (TIDAR) are affected: female mice exposed to PFOA or PFOS exhibited significant IgM reductions (Peden-Adams et al. 2008; Dewitt et al. 2016), whereas male mice did not show similar changes (Qazi et al. 2010), suggesting sex-related differences. However, not all PFAS show consistent effects. For example, perfluorodecanoic acid (PFDA), PFHxS, and several methoxy-substituted perfluorinated acids did not significantly alter immunoglobulin levels (Frawley et al. 2018; Woodlief et al. 2021), highlighting variability across compounds and study designs.

In line with human epidemiology and in vivo animal studies, also in vitro studies have shown that PFASs can alter B-cell functions and Igs production. Our previous work and the EFSA report demonstrated that PFAS inhibit antibody release in human peripheral blood mononuclear cells (PBMCs) using TIDAR and TDAR models in a concentration-dependent manner (Corsini et al. 2024; Iulini et al. 2025; EFSA, 2020). These effects mirror some animal findings and strengthen the evidence for immunotoxicity. Mechanistic studies provide additional insight: PFAS can alter B-cell function and immunoglobulin production by modulating gene expression. Research on the effects of PFAS in the human Namalwa B lymphoma cell line revealed that PFNA, PFOA, PFHxS, and PFOS alter gene expression related to B cell development and primary immunodeficiency signaling, in particular decreasing recombination activating gene 1 (*RAG1*) and *RAG2* gene expression (Janssen et al. 2023). Of the tested substances, PFNA decreased *RAG1* and *RAG2* expression most potently. A subsequent study examining the effects of 15 PFASs on *RAG1* and *RAG2* gene expression in Namalwa cells and their activity in Jurkat interleukin 2 (IL-2) reporter T-cells, identified HFPO-TA and PFDA as the most potent of the tested PFAS, respectively (Janssen et al. 2024a). Overall, in vitro data indicate effects of PFASs on relevant aspects of antibody production by B cells, which can at least partly provide an explanation behind the observed reduction in vaccine efficacy in exposed populations.

Although effects of PFASs on antibody production were observed in many studies, the mechanisms behind the observed effects still remain unclear. A growing body of evidence suggests the disruption of nuclear receptor signaling to play a role in the observed effects, particularly involving the peroxisome proliferator-activated receptor alpha (PPARα) and the glucocorticoid receptor (GR) as possible underlying mechanisms (Corsini et al. 2011; Ehrlich et al. 2023; Masi et al. 2022). Both receptors play critical roles in immune regulation, lipid metabolism, and inflammatory responses (Baschant & Tuckermann 2010; Christofides et al. 2022). PFASs have been shown to act as ligands or modulators of these receptors, thereby altering the expression of genes involved in immune cell activation, proliferation, and cytokine production (Corsini et al. 2024; Janssen et al. 2023; Masi et al. 2022). Yet, critical gaps remain in our understanding of the specific molecular mechanisms involved in these different endpoints. To address some of these knowledge gaps, in this study, the effects of PFOA and PFOS exposure in human PBMCs were characterized using RNA sequencing (RNAseq). The experimental protocol followed was previously used to study the effects of PFASs on B lymphocyte activation and antibody secretion (Corsini et al. 2024; Iulini et al. 2025). Analyses were performed after 24 h and after 7 days of exposure. The involvement of the GR in the effects of PFOA, PFOS, PFNA, and PFHxS leading to reduced antibody secretion was verified.

## Materials and methods

### Chemicals and reagents

PFOA (CAS #335-67-1, purity 95%), PFOS (CAS #1763-23-1, acid solution ~ 40% in H_2_O), PFNA (CAS #375-95-1; purity 97%), PFHxS (CAS #3871–99-6, purity ≥ 98%), dexamethasone (DEX, 9α-fluoro-16α-methylprednisolone, CAS #50-02-2, purity ≥ 98%), mifepristone (CAS # 84,371-65-3, purity ≥ 98%) and rapamycin (CAS # 53,123-88-9, purity ≥ 95%) were purchased from Sigma-Aldrich (St Louis, MO, USA). All the chemicals were dissolved in dimethyl sulfoxide (DMSO, CAS #67-68-5). Recombinant human IL-2 (hIL-2) was purchased from Miltenyi Biotec (Bergisch Gladbach, Germany), Class B CpG oligodeoxynucleotide (ODN) 2006 (ODN 7909) was purchased from InvivoGen (San Diego, CA, USA) and both were dissolved in Dulbecco’s phosphate-buffered saline (PBS).

### PBMC isolation

PBMCs were isolated from buffy coats obtained from anonymous male and female healthy donors purchased from the Niguarda Hospital (Milan, Italy). PBMCs were isolated by Ficoll gradient centrifugation and subsequently washed five times with PBS. For the experiments, PBMCs were suspended in RPMI 1640 without phenol red containing 2 mM L-glutamine, 100 IU/mL penicillin, 0.1 mg/mL streptomycin, 10 µg/mL gentamycin, 50 µM 2-mercaptoethanol, supplemented with 5% of heat-inactivated human serum (hereafter referred to as complete medium). The aforementioned cell culture medium, serum and all supplements were purchased from Sigma-Aldrich. Cells were incubated in a humidified atmosphere with 5% CO_2_ at 37 °C.

Concentrations of PFOA, PFOS, PFNA, and PFHxS cytotoxic to PBMCs were determined using a CyQUANT™ Lactate Dehydrogenase (LDH) Cytotoxicity Assay Kit (InvitrogenTM Corporation, MA, USA) and according to the manufacturer’s instructions (data already published by (Corsini et al. 2024). Only non-cytotoxic concentrations were used in subsequent experiments.

### PBMC exposure for RNA sequencing

For RNA sequencing, two independent experiments were conducted in which PBMCs were exposed to PFOA and PFOS.

In both experiments PBMCs were isolated from 5 male and 5 female donors and seeded in 48-wells plates containing 1.26 × 10^6^ PBMCs in 1 mL of complete medium. In the first independent experiment, PBMCs were exposed to PFOA or PFOS (at the concentration of 12.6 µg/mL, corresponding approximately to 25 µM PFOS and 30 µM PFOA), and DMSO as vehicle control (final concentration 0.1% (v/v)) for 24 h. Following the protocol by Tuijnenburg et al. (2020), the cells were subsequently stimulated with 100 IU/mL of rhIL-2 and 1 μg/mL ODN2006 for 6 days to induce B cell differentiation and activation (hereafter referred to as stimulated PBMCs). During stimulation, the PBMCs remained exposed to PFAS, resulting in a total exposure duration of seven days. In the second experiment, PBMCs were exposed either to PFOA or PFOS (at the concentration of 12.6 µg/mL), DEX (150 µg/mL) or DMSO as vehicle control (final concentration 0.1% (v/v)) and incubated for 24 h without subsequent stimulation (hereafter referred to as PBMCs without subsequent stimulation).

### RNA isolation

Following exposure, RNA was extracted using RNeasy Mini spin columns (Qiagen, Hilden, Germany) according to the manufacturer’s instructions. Briefly, suspension and attached cells were collected after gentle scraping, centrifuged at 200 × *g* for 7 min, following removal of the supernatant, the cells were lysed using RTL buffer containing β-mercaptoethanol (1:100). The lysate was then transferred into an RNAse-free microcentrifuge tube and vortexed at maximum speed for 1 min. Subsequently, an equal volume of 70% ethanol (Sigma-Aldrich) was added, mixed, transferred onto RNeasy spin columns, and centrifuging at 9500 × *g* for 30 s. To eliminate residual buffer, 350 μL of Buffer RW1 was added, followed by centrifugation at 9500 × *g* for 30 s. The flow-through was discarded. For on-column DNase digestion, 80 μL of DNase solution (prepared by mixing 10 μL of DNase stock solution with 70 μL of Buffer RDD) was applied to the membrane and incubated at room temperature for 15 min. Subsequently, 350 μL of RW1 Buffer was added to the column and centrifuged at 9500 × *g* for 30 s for the RNA wash step. The flow-through was discarded. A second wash was performed using 500 μL RPE Buffer per column, followed by a 30-s centrifugation. This process was repeated with 2 min of centrifugation. After that, the column was subjected to additional centrifugation for 1 min at maximum speed to dry the membrane. Finally, the column was transferred into a new 1.5 mL RNase-free microcentrifuge tube, and 30 μL of RNase-free water was added for the final RNA elution. RNA was collected after 1 min of centrifugation at 9500 × *g*. The isolated RNA was stored at − 80 °C.

RNA concentration and purity were determined by measuring the absorbance of the samples at 230, 260, and 280 nm using a NanoReady F-3100 spectrophotometer (Life Real, Zhejiang, China). The optical density (OD) ratios A260/A280 and A260/A230 were used to assess RNA purity.

### RNA seq analysis

#### Library preparations and RNA sequencing

To obtain insight into the cellular and molecular effects of 7 day PFASs exposure in stimulated PBMCs and after 24-h exposure to PFOA and PFOS in unstimulated PBMCs, transcriptomic analysis was performed. RNA integrity was assessed using a total RNA Pico chip in an Agilent 2100 Bioanalyzer (Agilent Technologies). For RNA sequencing, RNA samples were sent to Azenta Life Sciences (Leipzig, Germany). At the sequencing facility, following quality assessment and poly(A) selection, library preparation was performed using the NEBNext Ultra II Directional RNA Library preparation kit. Samples were sequenced on a Illumina NovaSeq platform using paired-end reads (2 × 150 bp length), generating at least 12 Gb per sample. RNA sequencing quality met the threshold of at least 80% of the bases with a Phred score of ≥ Q30. RNAseq data have been deposited in the NCBI Gene Expression Omnibus under accession number GSE311234 for stimulated PBMCs (7-day exposure) and GSE311566 for PBMCs without subsequent stimulation (24-h exposure).

#### Processing of RNA sequencing reads

Details on the processing of RNA sequencing reads have been extensively described previously (Janssen et al. 2024b). The reads were aligned to the transcriptome sequences based on the GRCh38.p13 (release 42) human genome assembly, as annotated by the GENCODE consortium using HISAT2 (version 2.2.1) (Kim et al. 2019).

#### RNA sequencing data analysis

Differential gene expression data, generated using Limma (version 3.50.3) (Ritchie et al. 2015), were visualized with volcano plots, displaying a statistical significance threshold of *p*-value < 0.01. Further insights were obtained through Ingenuity Pathway Analysis (IPA; Version 84,978,992; Qiagen, Redwood City, CA, USA). For analysis of the differential gene expression data from both stimulated PBMCs and from PBMCs exposed for 24 h without subsequent stimulations in IPA, the following criteria were applied for inclusion of molecules in the analysis: for PBMCs derived from female donors, a *p*-value < 0.05; and for PBMCs derived from male donors, a log2 fold change > 0.5 or < -0.5 (corresponding to a fold change of > 1.4 or < 0.71) combined with a *p*-value < 0.05. To uncover potential upstream mechanisms driving the differential gene expression upon PFAS exposure the Upstream Regulator analysis module in IPA was used. To assess overlap differentially expressed genes (*p* < 0.05) of PBMCs exposed to PFOA, PFOS and DEX for 24 h, Venn diagrams were generated using the open access tool Venny (version 2.1.0; https://bioinfogp.cnb.csic.es/tools/venny/). Additionally, normalized count data generated with DESeq2 were used for Geneset Enrichment Analysis (GSEA; version 4.3.2; Broad Institute, Cambridge, MA, USA) and for the generation of heatmaps. GSEA was used to identify enriched or suppressed gene sets (Subramanian et al. 2005). For analysis using GSEA, gene sets were derived from Biocarta, KEGG and WikiPathway databases and ranked based on the NES (cut-off nominal *p*-value < 0.05 and FDR q-value < 0.05). Only gene sets comprising more than 15 and fewer than 500 genes were taken into account. Statistical significance of GSEA results was determined using 1000 permutations. Heatmaps of differentially expressed genes in PBMCs following 24-h exposure to PFOS and PFOA were prepared using Morpheus (https://software.broadinstitute.org/morpheus/). Genes that were differentially expressed with a *p*-value < 0.05, determined using the empirical Bayed moderated t-statistic following DEX exposure, were included and compared to their expression in PBMCs exposed to PFOA and PFOS. Gene expression, relative to PBMCs exposed to DMSO control, were hierarchically clustered using the Euclidean distance algorithm.

### Verification of the involvement of GR in total IgG and IgM secretion

In this study, we aim to expand on the findings of Iulini (2025) by investigating whether the GR contributes to the reduction in antibody secretion induced by PFOA, PFOS, PFNA and PFHxS in stimulated PBMCs. To verify the involvement of GR, PBMCs isolated from 5 male and 5 female donors, were seeded at 1.26 × 10^6^ cells/mL in a 48-well plates in complete medium. Cells were exposed to the GR-antagonist mifepristone at 10 μM for 1 h (Corsini et al. 2014; Masi et al. 2022) before the addition of PFOA, PFOS, PFNA, and PFHxS at 12.6 µg/mL, DEX at 150 µg/mL or DMSO as vehicle control (final concentration 0.2% (v/v)) for 24 h. After the incubation period, PBMCs were then stimulated to induce B cell differentiation and activation with 1 µg/mL of ODN2006 and 100 IU/mL of rhIL-2 for 6 days.

To determine the release of total IgG and IgM, after a total of 7 days, PBMCs were collected after gentle scraping and centrifuged at 25 °C at 200 × *g* for 5 min. Supernatants were collected and stored at -20°C until further analysis. Levels of total IgM and IgG in the medium were measured using a custom enzyme-linked immunosorbent assay (ELISA) that was assembled in-house, using individual reagents from Sigma-Aldrich as previously described (Corsini et al. 2024; Iulini et al. 2025). Briefly, 100 µL of anti-human IgG (Cat. No. I1886) and/or anti-human IgM (Cat. No. I0104, Sigma-Aldrich) solution at 1 µg/mL in PBS were plated in a 96-well plate and incubated overnight at 4°C. Subsequently, 100 µL of standard (0–1000 ng/ml, IgG from human serum Cat. No. I4506, IgM from 160 human serum I8260) or samples properly diluted in reagent buffer (PBS + 0.5% of bovine serum albumin + 0.05% of Tween 20) were added and incubated for 2 h at room temperature. Then, 100 µL of anti-human polyvalent Igs (Cat. No. A3313) diluted 1:5000 in reagent buffer was added and incubated for 1 h at room temperature. Finally, 100 μL of Phosphatase substrate 4 mg/mL (CAS # 333,338–18-4) diluted in alkaline phosphatase (AP) buffer (composed by Tris CAS # 77–86-1, NaCl CAS # 7647–166 14–5, MgCl2•6H_2_O CAS # 7791–18-6, NaOH CAS # 1310–73-2 and H_2_O) were added and absorbance was read at 415 nm. Data were analyzed using SoftMax Pro 7.1.2. Results are expressed as fold-change of chemical-treated cells *versus* vehicle-treated cells (DMSO) and reported as mean ± standard error of mean (SEM) of 5 male and 5 female independent donors. Statistical analysis was performed using GraphPad Prism version 10.2.3 (GraphPad Software, La Jolla, CA, United States). Significant differences were determined using a one-way ANOVA, followed by Dunnet’s test or a paired T-test, as indicated in the figure legends. Results were considered significant if *p* < 0.05.

## Results

### Cell viability

To ensure that only non-cytotoxic concentrations were employed in subsequent experiments, the leukotoxicity after increasing concentration of PFOA, PFOS, PFNA, and PFHxS on PBMCs was assessed using a CyQUANT™ LDH Cytotoxicity Assay Kit according to the manufacturer’s instructions (data previously reported by (Corsini et al. 2024 and Iulini et al. 2025).

### Transcriptomic analysis of stimulated PBMCs exposed to PFOA and PFOS

**Fig. 1:**
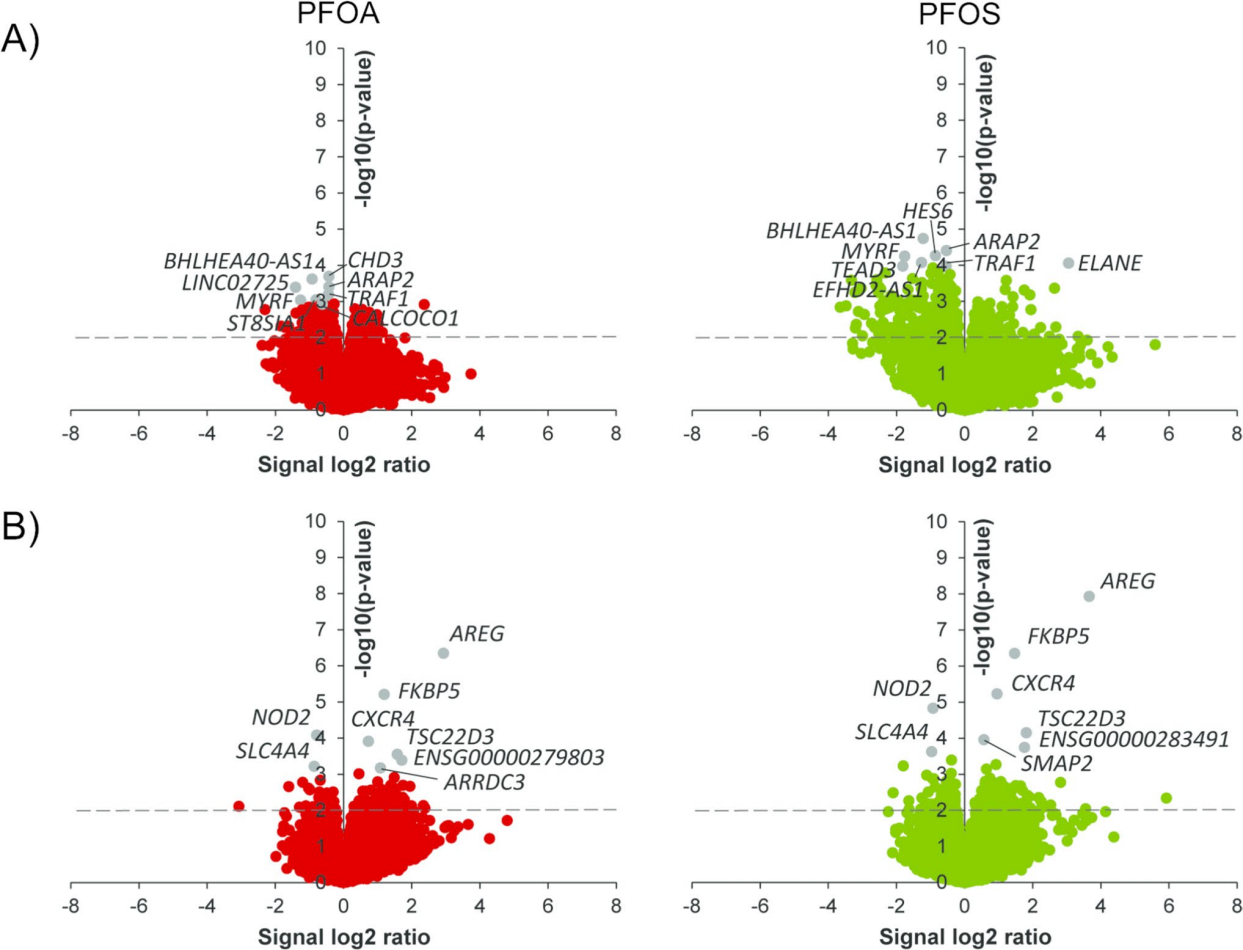
Differentially expressed genes in stimulated PBMCs obtained from **A** five male and **B** five female healthy donors after 7-day exposure to 12.6 µg/mL PFOA or PFOS. Changes in gene expression following exposure to the test chemicals compared to solvent control (expressed as signal log2 ratio, x-axis) are plotted against statistical significance (expressed as -log 10 *p*-value of empirical Bayes moderated t-statistic *p*-value, y-axis). The dotted line indicates the statistical significance threshold set at *p* < 0.01. The 8 most significantly regulated genes are depicted in grey

To obtain insight in the effects of PFOA and PFOS exposure on human PBMCs, genome-wide transcriptomics analysis was performed using RNA sequencing. To that end, a protocol in which PFOA and PFOS have previously been shown to reduce antibody release, including IgM and IgG was followed (Corsini et al. 2024; Iulini et al. 2025). PBMCs were exposed to 12.6 µg/mL PFOA and PFOS for a duration of 24 h, before differentiation and activation of B lymphocytes. Exposure to PFOA significantly (*p* < 0.01) affected the expression of 200 genes, and PFOS altered the expression of 414 genes in PBMCs obtained from male donors (Fig. 1A). In PBMCs obtained from female donors, fewer genes were affected, with PFOA significantly altering the expression of 100 genes and PFOS of 179 genes (Fig. 1B). Overall, in both sexes, PFOS altered the expression of more genes than PFOA. The eight most significantly regulated genes in each condition are highlighted in Fig. 1 and are listed in Table 1. Nearly all of the eight most significantly regulated genes in PBMCs obtained from male donors were downregulated by both PFOA and PFOS, except for a single gene, *ELANE*, which was upregulated by PFOS. In contrast, in PBMCs from female donors, six of the eight most significantly regulated genes were upregulated after PFOA and PFOS exposure, while the remaining two genes, *NOD2* and *SLC4A4*, were downregulated by both compounds.

**Table 1:** A. The eight most significantly regulated genes in stimulated PBMCs from male donors exposed to 12.6 µg/mL PFOA. B. The eight most significantly regulated genes in stimulated PBMCs from male donors exposed to 12.6 µg/mL PFOS. C. The eight most significantly regulated genes in stimulated PBMCs from female donors exposed to 12.6 µg/mL PFOA. D. The eight most significantly regulated genes in stimulated PBMCs from female donors exposed to 12.6 µg/mL PFOS

Gene symbol	Description	*p* value	Fold change	Ensembl gene ID
*(A)*				
*CHD3*	Chromodomain helicase DNA binding protein 3	2.03E-04	-1.34	ENSG00000170004
*BHLHE40-AS1*	BHLHE40 antisense RNA 1	2.39E-04	-1.89	ENSG00000235831
*ARAP2*	ArfGAP with RhoGAP domain, ankyrin repeat and PH domain 2	3.92E-04	-1.34	ENSG00000047365
*LINC02725*	Long intergenic non-protein coding RNA 2725	4.08E-04	-2.65	ENSG00000273415
*TRAF1*	TNF receptor associated factor 1	6.45E-04	-1.36	ENSG00000056558
*ST8SIA1*	ST8 alpha-N-acetyl-neuraminide alpha-2,8-sialyltransferase 1	9.15E-04	-1.74	ENSG00000111728
*MYRF*	Myelin regulatory factor	9.30E-04	-2.39	ENSG00000124920
*CALCOCO1*	Calcium binding and coiled-coil domain 1	1.14E-03	-1.45	ENSG00000012822
*(B)*				
*BHLHE40-AS1*	BHLHE40 antisense RNA 1	1.85E-05	-2.32	ENSG00000235831
*ARAP2*	ArfGAP with RhoGAP domain, ankyrin repeat and PH domain 2	3.98E-05	-1.45	ENSG00000047365
*HES6*	Hes family bHLH transcription factor 6	5.49E-05	-1.81	ENSG00000144485
*MYRF*	Myelin regulatory factor	5.65E-05	-3.39	ENSG00000124920
*EFHD2-AS1*	EFHD2 antisense RNA 1	8.55E-05	-2.40	ENSG00000228140
*ELANE*	Elastase, neutrophil expressed	8.75E-05	8.30	ENSG00000197561
*TEAD3*	TEA domain transcription factor 3	1.06E-04	-3.50	ENSG00000007866
*TRAF1*	TNF receptor associated factor 1	1.08E-04	-1.45	ENSG00000056558
*(C)*				
*AREG*	Amphiregulin	4.53E-07	7.63	ENSG00000109321
*FKBP5*	FKBP prolyl isomerase 5	6.23E-06	2.29	ENSG00000096060
*NOD2*	Nucleotide binding oligomerization domain containing 2	8.31E-05	-1.72	ENSG00000167207
*CXCR4*	C-X-C motif chemokine receptor 4	1.23E-04	1.66	ENSG00000121966
*TSC22D3*	TSC22 domain family member 3	2.85E-04	2.99	ENSG00000157514
*ENSG00000279803*	Uncategorized gene	4.09E-04	3.28	ENSG00000279803
*SLC4A4*	Solute carrier family 4 member 4	6.13E-04	-1.81	ENSG00000080493
*ARRDC3*	Arrestin domain containing 3	6.80E-04	2.12	ENSG00000113369
*(D)*				
*AREG*	Amphiregulin	1.18E-08	12.66	ENSG00000109321
*FKBP5*	FKBP prolyl isomerase 5	4.46E-07	2.77	ENSG00000096060
*CXCR4*	C-X-C motif chemokine receptor 4	5.92E-06	1.94	ENSG00000121966
*NOD2*	Nucleotide binding oligomerization domain containing 2	1.50E-05	-1.90	ENSG00000167207
*TSC22D3*	TSC22 domain family member 3	7.05E-05	3.52	ENSG00000157514
*SMAP2*	Small ArfGAP2	1.10E-04	1.49	ENSG00000084070
*ENSG00000283491*	Uncategorized gene	1.82E-04	3.39	ENSG00000283491
*SLC4A4*	Solute carrier family 4 member 4	2.36E-04	-1.95	ENSG00000080493

### IPA of stimulated PBMCs exposed PFOA and PFOS

**Fig. 2:**
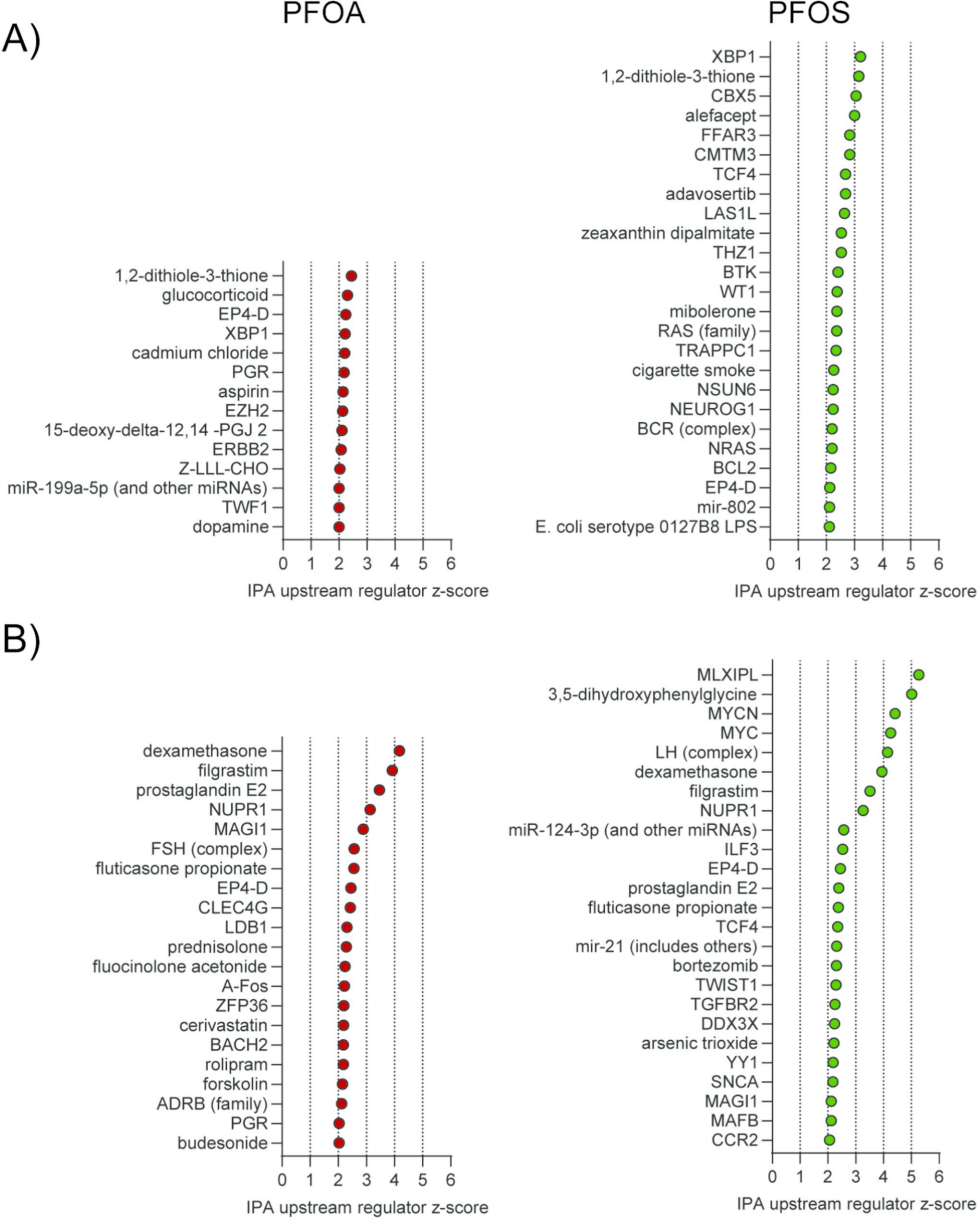
Upstream Regulator analysis by IPA of stimulated PBMCs obtained from **A** five male and **B** five female donors after 7-day exposure to 12.6 µg/mL PFOA or PFOS. The IPA upstream regulator z-score reflects the predicted activation state, with only positive values (Z-scores ≥ 2) shown here, indicating activated regulators considered biologically relevant. For PBMCs exposed to PFOS, the 25 upstream regulators ranked by positive Z-score are listed. For PMBCs exposed to PFOA, all the upstream regulators with a positive Z-score are shown. Only statistically significant Z-scores (*p*-value of overlap < 0.05) are included

To identify potential upstream mechanisms underlying the differential gene expression in stimulated PBMCs exposed to PFASs, IPA was applied using the Upstream Regulator analysis module. These ‘upstream regulators’ are molecules that are reported to cause comparable changes in, among others, gene expression. Consistent with the higher number of differentially expressed genes observed in activated PMBCs exposed to PFOS compared to PFOA, a higher number of potential upstream regulators with a positive Z-score were identified in response to PFOS exposure (Fig. 2). Furthermore, in activated PBMCs derived from female donors, the significant Z-scores of the upstream regulators had higher Z-scores than those observed in male donors. In activated PBMCs from female donors, PFOA exposure induced gene expression changes that most significantly overlapped with those of the GR agonist DEX (Z-score of 4.174; *p*-value of overlap: 9.5 × 10^–5^). Prednisolone, another glucocorticoid agonist, was also suggested as upstream regulator, with a Z-score of 2.283. Interestingly, glucocorticoids were also implicated as upstream regulator in activated PBMCs obtained from female donors exposed to PFOS (DEX, Z-score: 3.944) and from male donors exposed to PFOA (glucocorticoid, Z-score: 2.299). Moreover, several glucocorticoid receptor target genes, such as *AREG* (Wei et al. 2023), *TRAF1* (Wei et al. 2023), *CXCR4* (Otha 2000; Piovan et al. 2013; Cain et al. 2020), *TSC22D3* (Lambert et al. 2013; Piovan et al. 2013; D’Adamio et al., 1997; Cain et al. 2020; Van Moortel et al. 2022), *SMAP2* (Piovan et al. 2013) and *FKBP5* (Lambert et al. 2013; Gans et al. 2021; Van Moortel et al. 2022), were among the top eight most significantly differentially expressed genes in PBMCs from both genders following exposure to both PFOA and PFOS (Fig. 1; Table 1). The top eight most highly upregulated and downregulated genes upon exposure to PFOA and PFOS in stimulated PMBCs are listed in Supplementary Tables 1 and 2, respectively. Notably, CD163, a well-known GR-target gene (Högger et al. 1998; Galon et al. 2002), was identified as the most highly upregulated gene in stimulated PBMCs from male donors exposed to PFOS (fold-change of 48.4; Table S1B), as well as in activated PBMCs from female donors exposed to PFOA (fold-change of 27.9; Table S1C) and PFOS (fold-change of 60.4; Table S1D). Taken together, the gene expression changes in activated PBMCs exposed to PFOA and PFOS point towards involvement of GR-signaling. PFOS was found to enhance the expression of GR-target genes in activated PBMCs from both male and female donors, while PFOA had a more pronounced effect in activated PBMCs from female donors.

### GSEA of stimulated PBMCs exposed to PFOA and PFOS

GSEA was performed to gain further insights into the mechanisms affected by PFOA and PFOS exposure. Interestingly, PBMCs derived from male donors exhibited a higher number of induced gene sets following PFOA and PFOS exposure, whereas PBMCs from female donors showed a greater number of repressed gene sets. In stimulated PBMCs obtained from both sexes, PFOA and PFOS exposure affected various gene sets, including induction of ribosome-associated gene sets (Supplementary Figs. 1 and 2). Regarding repressed gene sets, in male donors, PFOA did not repress any, while PFOS repressed only one: natural killer cell mediated toxicity. In contrast, PBMCs from female donors exhibited repression of several gene sets (Supplementary Figs. 1 and 2). Among these, multiple gene sets related to cholesterol metabolism were downregulated by PFOA and PFOS, including ‘cholesterol metabolism with block and Kandutsch-Russel pathways’, ‘cholesterol synthesis disorders’, ‘cholesterol biosynthesis pathway in hepatocytes’ and ‘cholesterol biosynthesis pathway’. Notably, both PFOA and PFOS repressed the ‘B cell receptor signaling pathway’ gene set.

### Transcriptomic analysis of PBMCs exposed to PFOA, PFOS, and DEX for 24 h without subsequent stimulation

In the previously described transcriptomics analysis of activated PMBCs, cells were initially exposed to 12.6 µg/mL PFOA and PFOS for a duration of 24 h, after which ODN2006 and rhIL-2 were added for an additional 6 days to induce B lymphocyte differentiation and activation, resulting in a total exposure duration of 7 days. To gain insight into potential initiating events of PFASs on PBMCs, a second study was conducted in which PBMCs were exposed to 12.6 µg/mL PFOA and PFOS for 24 h, followed by transcriptomic analysis. Since the previous analysis of stimulated PBMCs pointed towards activation of glucocorticoid signaling by PFOA and PFOS, an additional exposure to the GR agonist DEX was included for comparison.

**Fig. 3:**
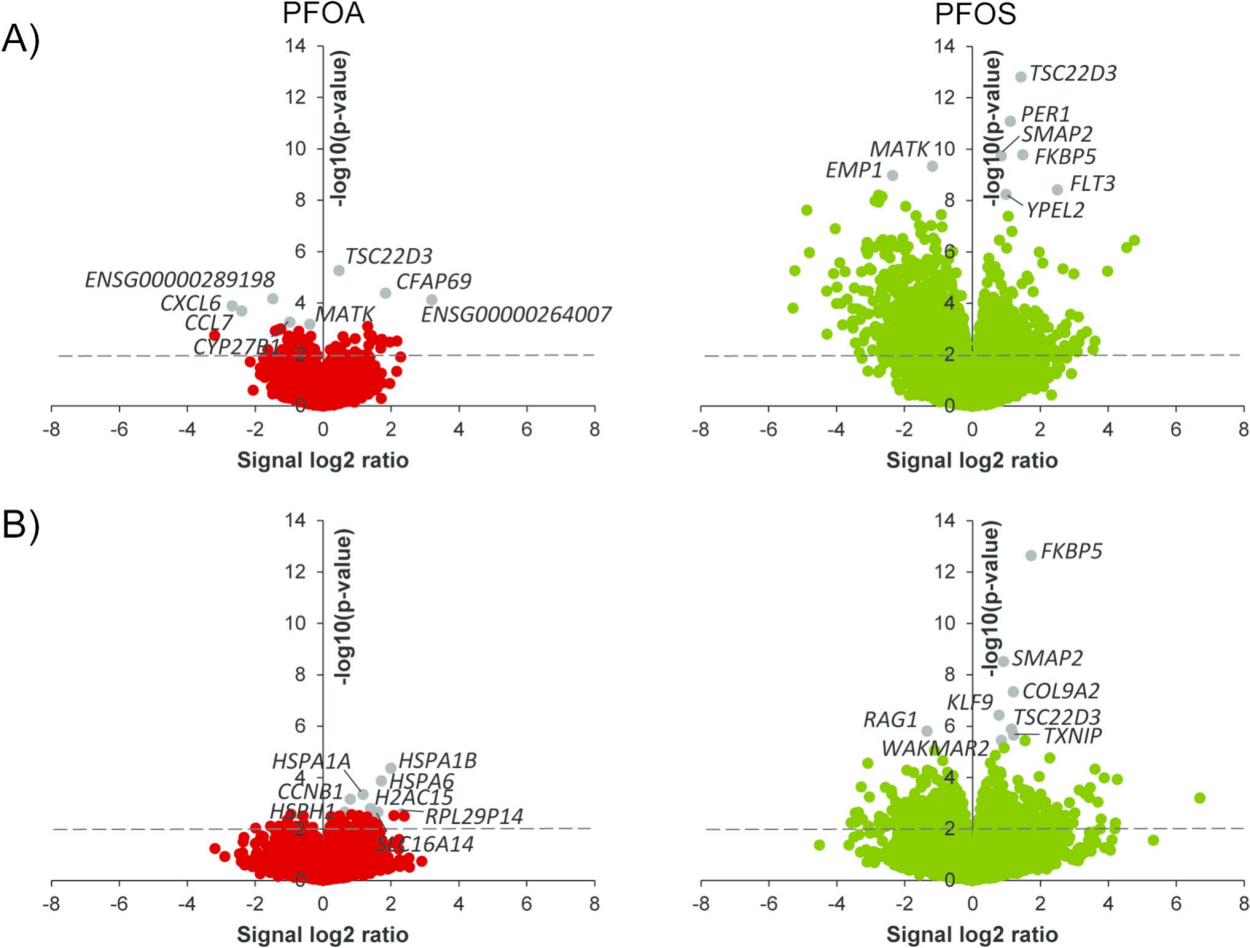
Differentially expressed genes in PBMCs obtained from (A) five male and (B) five female donors after 24 h exposure to 12.6 µg/mL PFOA or PFOS. Changes in gene expression following exposure to the test chemicals compared to solvent control (expressed as signal log2 ratio, x-axis) are plotted against statistical significance (expressed as -log 10 *p*-value of empirical Bayes moderated t-statistic *p*-value, y-axis). The dotted line indicates the statistical significance threshold set at *p* < 0.01. The 8 most significantly regulated genes are depicted in grey

Similar to the 7-day exposure of stimulated PBMCs, 24 h exposure of PBMCs to PFOA and PFOS also resulted in a higher number of differentially expressed genes (*p* < 0.01) in male donors compared to female donors. Also here, PFOS affected the expression of more genes than PFOA in both sexes. In PBMCs obtained from male donors, PFOA affected the expression of 58 genes, while PFOS altered the expression of 1251 genes (Fig. 3A). In PBMCs obtained from female donors, PFOA altered the expression of 43 genes and PFOS 688 genes (Fig. 3B). The top eight most significantly regulated genes are highlighted in grey in Fig. 3. Supplementary Tables 3, 4 and 5 provide details on the eight most significantly regulated, most highly upregulated, and most highly downregulated genes, respectively. Several well-known GR-target genes, including *FKBP5, TSC22D3 and ADORA3,* were among those most affected genes, particularly in response to PFOS. In both sexes, the most significantly altered genes in PBMCs exposed to PFOS were GR-target genes, being *TSC22D3* in PBMCs from male donors (Fig. 3A; Supplementary Table 3B) and *FKBP5* in PMBCs from female donors (Fig. 3B; Supplementary Table 3D).

**Fig. 4:**
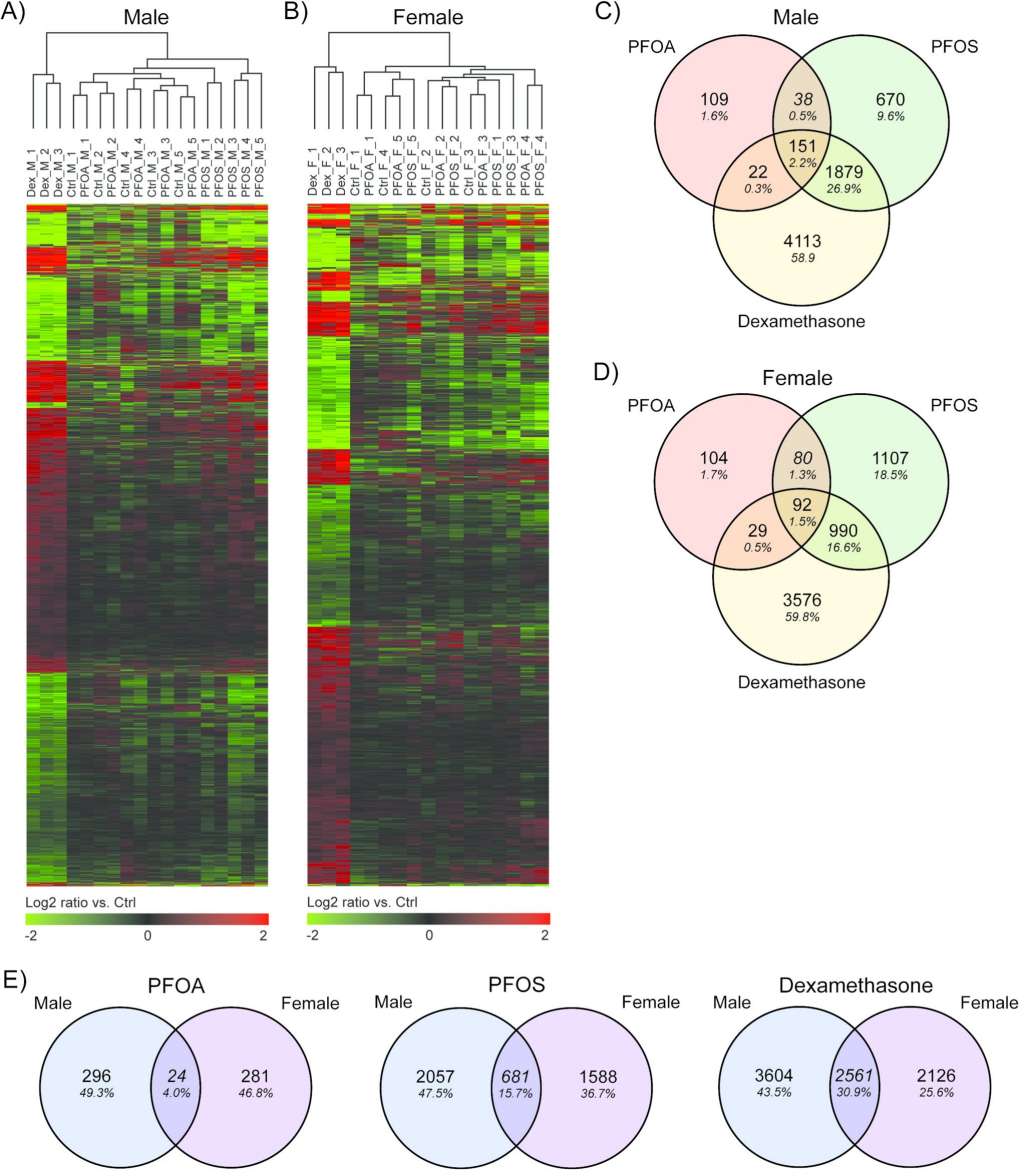
Differentially expressed genes in PBMCs obtained from male and female donors after 24 h exposure to 12.6 µg/mL PFOA, 12.6 µg/mL PFOS, and 150 µg/mL DEX. The heatmaps display the Log2 expression ratio of genes, significantly altered by DEX (p < 0.05), in PMBCs obtained from **A** male and **B** female donors exposed to PFOA, PFOS and DEX compared to vehicle control DMSO (Ctrl). Gene expression in individual samples is compared to the average expression in the vehicle control (which is set to ‘zero’). Hierarchical clustering was performed using Euclidean distance. Venn diagrams displaying the overlap of differentially expressed genes (p < 0.05) in PBMCs from **C** male and **D** female donors following exposure to PFOA, PFOS and DEX. **E** Venn diagrams displaying the overlap of differentially expressed genes (p < 0.05) following PFOA, PFOS and dexamethasone exposure in PBMCs derived from male and female donors

To compare gene expression profiles of PBMCs exposed to PFOA and PFOS with PBMCs exposed to DEX, differentially expressed genes in PBMCs exposed to DEX were selected using a statistical cut-off of *p* < 0.05, which were 6165 genes in PBMCs from male donors and 4687 genes in PBMC from female donors. For these genes, the expression was compared for PBMCs exposed to DEX, PFOA, and PFOS (Fig. 4A, B, respectively). While overall the samples treated with DEX clustered separately from PFAS, also some overlapping patterns could be observed. Subsequently, the overlap of significantly differentially expressed genes (*p* < 0.05) of PBMCs exposed to PFOA, PFOS and DEX was analyzed (Fig. 4C, D). There was a greater overlap in significantly altered genes between PFOS and DEX, with 29.1% of all differentially expressed genes among PFOS, PFOA and DEX in male donors, and 18.1% in female donors, compared to the overlap between PFOA and DEX. Interestingly, comparison of differentially expressed genes between male and female PBMCs revealed only a small overlap between male and female PBMCs following PFOA exposure (4.0%), whereas PFOS exposure resulted in a greater overlap (15.7%). In contrast, dexamethasone induced a substantial shared transcriptomic response between sexes, with 30.9% overlap in differentially expressed genes (Fig. 4E).

### IPA of PBMCs exposed to PFOA, PFOS, and DEX for 24 h without subsequent stimulation

**Fig. 5:**
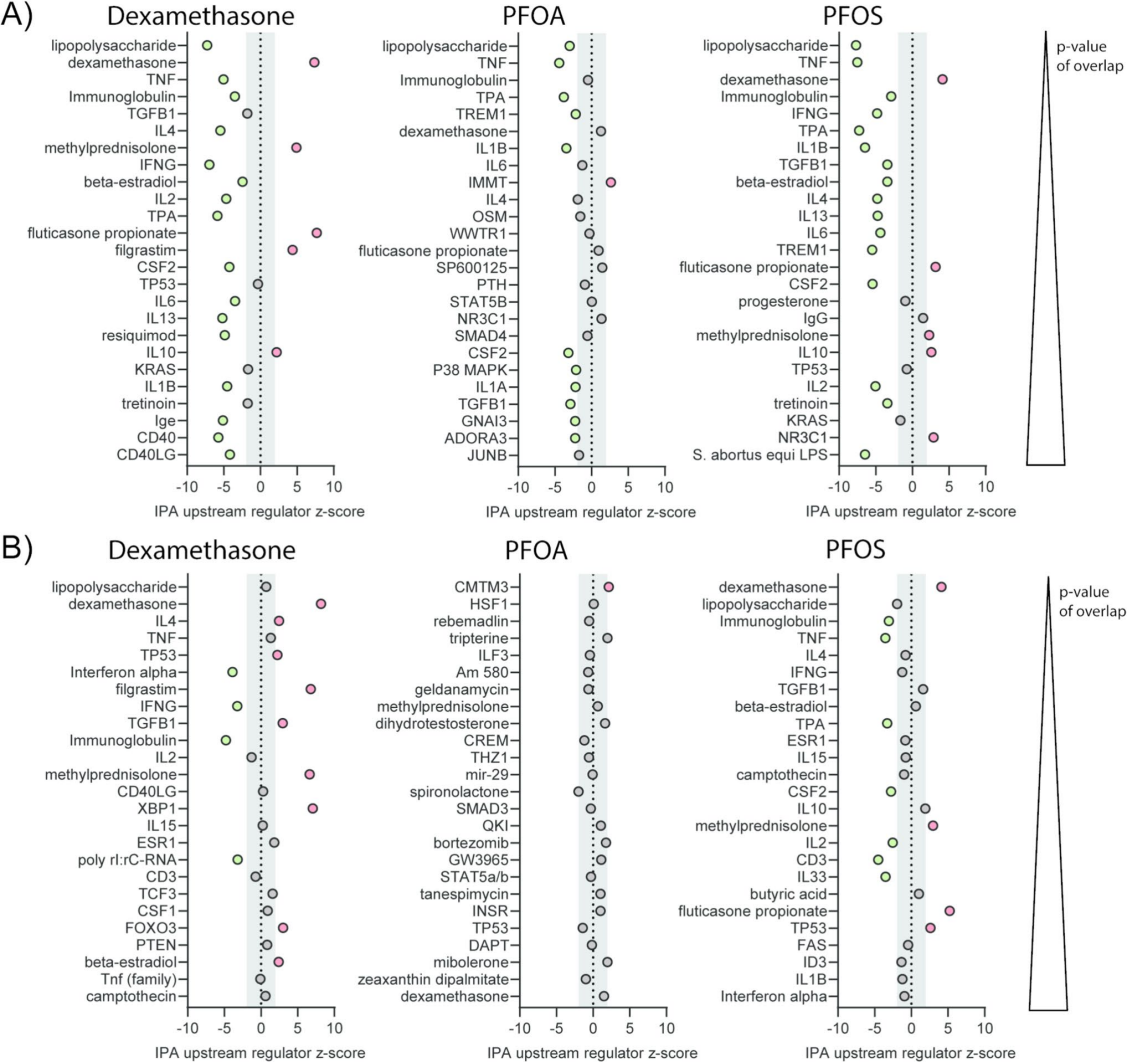
Z-scores of the top 25 predicted upstream regulators (ranked by lowest *p*-value of overlap) in PBMCs from **A** male and **B** female donors following 24 h exposure to 12.6 µg/mL PFOA, 12.6 µg/mL PFOS and 150 µg/mL DEX. Upstream regulators with the most significant *p*-values are listed at the top of the graph for each exposure. The z-score reflects the predicted activation state of each upstream regulator. Red dots represent a positive z-score (> 2), suggesting activation based on concordance between observed gene expression and known regulatory gene targets. Green dots indicate a negative z-score (< –2), indicating an inverse relationship between the observed gene expression and known regulatory gene targets. Grey dots within the grey area represent upstream regulators with z-scores between –2 and 2, and are considered to be not biologically relevant

To better understand potential initiating events following the exposure of DEX, PFOA, and PFOS to PBMCs, and to compare these between the treatments, the Upstream Regulator analysis module in IPA was used. Overall, for DEX more upstream regulators had a z-score below -2 and above 2, followed by PFOS and PFOA. In DEX- and PFOS-exposed PBMCs, DEX and methylprednisolone, which is also a GR agonist, were suggested as upstream regulators (Fig. 5). In PFOA-exposed PBMCs, these upstream regulators failed to meet the threshold of biological significance (i.e. a z-score below -2 or above 2). Comparison of the z-scores of the top 25 predicted upstream regulators, ranked by the lowest *p*-value of overlap, for PBMCs exposed to PFOA, PFOS, and DEX revealed high similarity between those suggested in PBMCs exposed to PFOS and those following DEX treatment. In PBMCs from male donors, 20 out of the 25 identified upstream regulators overlapped between PFOS and DEX treated cells, all showing regulation in the same direction between the treatments. In female donors, 16 out of the 25 identified upstream regulators overlapped between these two treatments, with 10 showing the same direction of regulation. In comparison, PFOA-treated PBMCs showed fewer overlapping upstream regulators with DEX-treated PBMCs (11 in male donors and only 2 in female donors), and the associated z-scores were substantially lower. Taken together, transcriptomic analysis of both activated PBMCs (7 day exposure) and PBMCs (24 h exposure without subsequent stimulation) points toward activation of the GR and GR-mediated signaling upon treatment with the tested PFASs (albeit at a different extent), with both PFOS and PFOA showing the most pronounced effects in stimulated PBMCs from female donors and PFOS in PBMCs from male donors.

### Role of GR in PFAS-induced suppression of immunoglobulin secretion

To verify if the reduction in antibody secretion by PFOA, PFNA, PFOS, and PFHxS, as previously described (Corsini et al. 2024; Iulini et al. 2025), is mediated via glucocorticoid signaling as is indicated for PFOA and PFOS by the transcriptomic analyses presented in this manuscript, a follow-up study was conducted. PBMCs obtained from healthy males and females were treated with the GR antagonist mifepristone (Castinetti et al. 2010) for 1 h, before exposure to PFOA, PFNA, PFOS, PFHxS and DEX for 24 h. Subsequently, cells were stimulated with ODN2006 and rhIL-2 to induce B cell differentiation and activation for 6 days. Thereafter, levels of total IgG and IgM were quantified in cell culture medium.

**Fig. 6:**
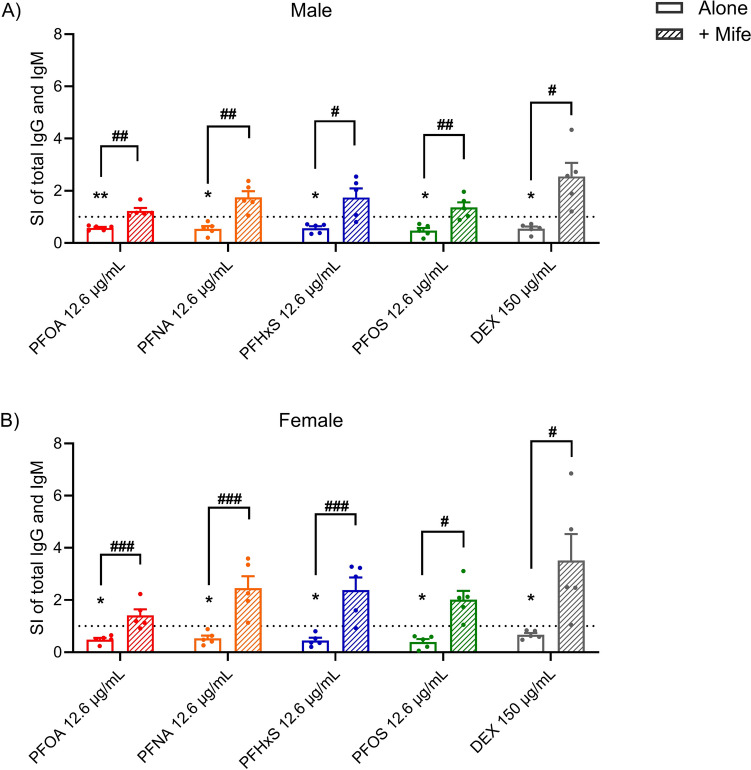
Role of GR in PFAS-induced antibody response suppression. Analysis of IgG and IgM released into cell culture medium after the treatment with 10 µM mifepristone for 1 h, followed by 12.6 µg/mL PFOA, PFNA, PFHXS and PFOS treatment in PBMCs obtained from 5 male **A** and 5 female **B** human donors. This was also done for the known glucocorticoid agonist DEX (150 µg/mL) for comparison. After 24 h, cells were stimulated with 1 µg/mL of ODN2006 and 100 U/mL of IL-2 to induce the B cell differentiation and activation. After 7 days, the concentrations of total IgG and IgM were quantified in the media. The results are expressed as SI of total IgG and IgM with respect to relative controls (cells exposed to vehicle or treated with vehicle + mifepristone). The columns represent the means, the whiskers the SEM, and the dots the individual values from the single donors. Statistical analysis was carried out using one-way ANOVA, followed by Dunnett’s test for PFAS vs vehicle control DMSO (represented by the dotted line set to 1) with *p < 0.05, **p 0.01, and paired t-test for PFAS alone vs PFAS + mifepristone, with # *p* <  0.05, ## *p* < 0.01, ### *p* < 0.001. The dotted line represents the control value set at 1

All four PFASs significantly reduced the release of total IgG and IgM in PMBCs from both male (Fig. 6A) and female (Fig. 6B) donors. Exposure to DEX also led to a decrease in total IgG and IgM secretion, underscoring the role of glucocorticoid signaling in the suppression of antibody production In the presence of mifepristone, the inhibitory effects of PFASs and DEX on antibody secretion were completely reversed, restoring total IgM and IgG release to levels comparable to or even higher than the control conditions of stimulated PBMCs exposed to vehicle control plus mifepristone, demonstrating the role of the GR in the PFAS-mediated inhibition of antibody secretion.

## Discussion

The immunosuppressive effects of PFASs are supported by substantial evidence from both human epidemiological studies and experimental models. A consistent and central finding is the association between PFAS exposure and reduced vaccine-induced antibody titres, particularly in pediatric and adolescent populations (Grandjean et al. 2012; Granum et al. 2013; Abraham et al. 2020). These studies, identified by EFSA as critical for deriving the health-base guidance value, provide robust evidence of immunosuppressive effects in infants and young children, an age group that appears to be particularly vulnerable to PFAS-mediated reductions in vaccine-induced antibody response and overall impairment of humoral immunity. Functionally, both TDAR and TIDAR responses are susceptible to PFASs interference, with reductions in serum IgM and IgG documented across multiple species and experimental models (Peden-Adams et al. 2008; Abraham et al. 2020; Antoniou et al. 2022). In vitro studies have further corroborated these findings, showing that exposure to PFOS, PFOA, PFNA and PFHxS suppresses immunoglobulin release in human PBMCs (Corsini et al. 2024; Iulini et al. 2025). In the present work, all four PFASs consistently reduced IgM and IgG secretion at 12.6 µg/mL in PBMCs from both male and female donors. This uniform suppression contrasts with the findings reported by Corsini et al. (2024), where statistically significant effects were not observed for PFHxS in male and PFOA in female at the highest concentration tested. Such differences likely reflect donor-dependent variability, a well-recognized source of heterogeneity in primary immune-cell assays, and underscore the importance of sufficiently large and diverse donor cohorts when characterizing immunotoxic responses. In this context, the more pronounced statistical significance obtained in the current study should be interpreted as a consequence of the stronger and more consistent suppression across the donor set examined, rather than a direct quantitative comparison with previous datasets.

The PFASs concentrations applied in this in vitro study exceed those typically measured in human serum following environmental exposure. Population-based biomonitoring data indicate that serum levels generally fall within the ng/mL range, which is substantially lower than the nominal concentrations used here (EFSA 2020; Abraham et al. 2020). This discrepancy reflects a common challenge in in vitro toxicology: experimental systems do not fully replicate physiological conditions, particularly with respect to compound bioavailability. Strong protein binding, limited cellular uptake, and the intrinsically acute nature of in vitro exposure paradigms contrast sharply with the chronic, lifelong exposure experienced by humans (Fischer et al. 2024). These considerations are especially relevant for sensitive developmental windows, including infancy and adolescence, during which epidemiological studies have repeatedly reported PFAS-associated reductions in vaccine-induced antibody responses (Grandjean et al. 2012; Granum et al. 2013; Abraham et al. 2020). Nonetheless, immunosuppressive effects at considerably lower, environmentally relevant concentrations have also been documented (Corsini et al. 2024), strengthening the biological plausibility of the effects observed in this study.

Although associations between human PFASs serum levels, and reduced antibody secretion have been observed in multiple epidemiological studies (Grandjean et al. 2012; Granum et al. 2013; Abraham et al. 2020), the underlying mechanisms remain incompletely understood. Evidence from in vitro studies point to direct interference of PFASs with key B- and T-cell processes. For instance, recent studies have shown that PFASs downregulate *RAG1* and *RAG2* gene expression in human Namalwa B lymphoma cells (Janssen et al. 2023; 2024a), an effect that could compromise V(D)J recombination and thereby the generation of a diverse repertoire of immunoglobulins (Sadofsky 2001). Furthermore, animal studies consistently show that PFOS and PFOA reduce TDAR, a benchmark indicator of immunotoxicity (DeWitt et al. 2008; Peden-Adams et al. 2008). At the molecular level, multiple immunomodulatory pathways are implicated in PFASs action. Increasing attention has focused on GR signaling as a potential mechanistic driver. GR activation is well established to suppress adaptive immune responses by inhibiting B-cell proliferation, antigen presentation, and cytokine output (Baschant & Tuckermann 2010). PFOS and PFOA have been shown to activate canonical GR-responsive genes, mimicking the effects seen with DEX or endogenous glucocorticoids (Corsini et al. 2024; Lambert et al. 2013; Gans et al. 2021; Van Moortel et al. 2022). In the present study, the upregulation of *TSC22D3* and *FKBP5* in PFAS-treated PBMCs and stimulated PBMCs was observed, which is in line with established steroid-responsive gene signatures. Particularly, *TSC22D3*, a GR-mediated gene involved in immune regulation, was upregulated not only in stimulated PBMCs exposed to PFASs, but also differentially regulated in Namalwa human B lymphoma cells treated with PFOA (Janssen et al. 2023). Similarly, RAG1, essential for immunoglobulin gene rearrangement during B cell development, was downregulated in female PBMCs exposed to PFOS, mirroring observations in Namalwa cells (Janssen et al. 2023). Gene-expression of FKBP5, a co-chaperone that interacts with, amongst others, heat shock protein 90 to regulate intracellular glucocorticoid signaling (Binder 2009), was significantly upregulated in stimulated PBMCs from male donors, indicating more changes related to the glucocorticoid-receptor. In addition, in silico modeling suggests PFOS can directly bind to the GR ligand-binding domain (Masi et al. 2022), reinforcing a structural mechanistic link to the observed immunotoxicity. The functional relevance of this pathway was supported by experiments with mifepristone, a GR antagonist, which partially prevented PFAS-induced suppression of IgG and IgM secretion. The increase above control values can be explained by the observation that blocking GR activation in B cells is associated with enhanced antibody production by enhancing B cell proliferation, differentiation into plasma cells, and immunoglobulin production (Kovacs 2014). Interestingly, in some donors, PFAS exposure in the presence of the GR antagonist resulted in higher antibody levels than PFAS alone, indicating inter-individual differences in GR-related feedback responses. Crucially, these findings highlight the mechanistic importance of GR signaling, and, by revealing donor-dependent variability, underscore the critical need for studies in larger, diverse donor cohorts.

The observed PFAS-induced activation of GR signaling and upregulation of GR-associated genes, in relation to the suppression of antibody secretion, can be more clearly interpreted by considering the regulatory role of FKBP5, a multifunctional immunophilin involved in several immune-regulatory pathways. FKBP5 is a gene encoding FKBP51, a 51 kDa co-chaperone that interacts with heat shock protein 90 and modulates the activity of multiple signaling proteins, including, but not limited to, the GR. Within the GR complex, FKBP5 reduces receptor affinity for glucocorticoids and delays GR nuclear translocation, thereby attenuating GR-mediated transcription (Zannas et al. 2016). Upon ligand binding, FKBP5 is exchanged for FKBP4, which promotes efficient GR nuclear translocation and transcriptional activity. Importantly, FKBP5 is itself a transcriptional target of GR, creating a well-described autoregulatory feedback loop. Beyond its role in GR signaling, FKBP5 has also been implicated in immunomodulatory pathways that are independent of glucocorticoid action. Increased expression of FKBP51 has been associated with reduced nuclear factor kappa-light-chain-enhancer of activated B cells (NF-κB) activity through impaired nuclear translocation of NF-κB and diminished transcriptional activity (Erlejman et al. 2014). While glucocorticoids are well known to suppress NF-κB signaling (Auphan et al. 1995; Nissen et al. 2000), similar inhibitory effects have also been reported for PFAS exposure (Corsini et al. 2011, 2012). In the current study, upstream regulator analysis following 24-h PFAS exposure of PBMCs revealed negative z-scores for NF-κB regulators, most predominantly for PFOS (Supplementary Table 6). This suggests that PFOS exposure may enhance GR signaling and suppress NF-κB activity, consistent with the glucocorticoid-mediated pathway described in adverse outcome pathway (AOP) 14 for immunosuppression, that could be mediated, at least in part, through FKBP5 induction. Supporting this observation, *CXCL6*, a chemokine strongly regulated by NF-κB was among the most significantly downregulated genes following 24-h exposure of PBMCs to PFOA and PFOS in male donors, and to PFOS in female donors (Supplementary Table 5). While FKBP5 has also been reported to enhance NF-κB signaling through interaction with the IκB kinase complex in certain cellular contexts (Zannas et al. 2019), our data support a inhibitory effect on NF-κB activity following PFAS exposure. In addition to modulating NF-κB signaling, FKBP5 can also inhibit calcineurin, a phosphatase that is critical for the activation of nuclear factors of activated T-cells (NFAT). By preventing NFAT dephosphorylation and nuclear translocation, FKBP5 ultimately impairs T-cell activation and cytokine production, including IL-2, a key driver of lymphocyte proliferation and differentiation (Becknell et al. 2012). This mechanism closely aligns with AOP 154, which describes how calcineurin inhibition leads to the suppression of the TDAR (Komatsu et al. 2021). The observed PFAS-induced upregulation of FKBP5 in the current study therefore provides a mechanistic link between molecular initiating events and downstream impairment of adaptive immune function. Importantly, while AOP154 focuses on TDAR, the present findings suggest that similar FKBP5-mediated mechanisms may also extend to TIDAR, particularly in scenarios where B cells are directly exposed to PFASs. Suppression of IL-2 and IL-4 signaling, both of which are critical for B-cell proliferation, class switching, and antibody secretion, provides a plausible explanation for the reduced IgM and IgG secretion observed in this study. Collectively, these data support a model in which PFAS exposure activates GR signaling, resulting in FKBP5 induction, and subsequent immune suppression via several converging pathways, including inhibition of the calcineurin–NFAT axis and altered NF-κB-mediated inflammatory signaling.

## Conclusion

In summary, these findings support a model in which PFAS exposure impairs humoral immunity primarily through GR signaling and upregulation of *FKBP5*, leading to suppressed B-cell antibody secretion. Differences observed between this study and our earlier study (Corsini et al. 2024) point to donor variability and subtle methodological nuances. Additionally, the finding that GR antagonism reverses the PFAS-induced reduction in antibody secretion reinforces the central role for this receptor. Considering the inherent variability in human immune responses, the use of different donors for transcriptomic studies, B cell assays, and antibody secretion assessments introduces a level of biological heterogeneity. While reflecting the natural population diversity, this variability complicates direct comparisons and emphasizes the importance of studies involving sufficient numbers of donors and replicates to draw robust conclusions. Moreover, the transcriptomic and gene set enrichment analyses performed here further elucidate the molecular basis of PFAS-induced immunomodulation, pointing to steroid hormone metabolism and sex differences as important factors. Although higher doses in vitro call for cautious interpretation with respect to human exposures, these mechanistic insights significantly advance our understanding of PFASs immunotoxicity and inform future risk assessment and regulatory considerations.

## Supplementary Information

Below is the link to the electronic supplementary material.

Supplementary file1.

## Abbreviations

AOP: Adverse outcome pathway
AP: Alkaline phosphatase
CAS: Chemical abstracts service
DEX: Dexamethasone
DMSO: Dimethyl sulfoxide
ELISA: Enzyme-linked immunosorbent assay
EPA: Environmental protection agency
EFSA: European food safety authority
GR: Glucocorticoid receptor
GSEA: Gene set enrichment analysis
HFPO-TA: Hexafluoropropylene oxide dimer acid
hIL-2: Human Interleukin-2
Ig: Immunoglobulin
IPA: Ingenuity pathway analysis
LDH: Lactate dehydrogenase
ODN2006: Class B CpG oligodeoxynucleotide 2006
NF-κB: Nuclear factor kappa-light-chain-enhancer of activated B cells
PBMCs: Peripheral blood mononuclear cells
PBS: Phosphate-buffered saline
PFAS: Per- and polyfluoroalkyl substances
PFHxS: Perfluorohexane sulfonate
PFDA: Perfluorodecanoic acid
PFNA: Perfluorononanoic acid
PFOA: Perfluorooctanoic acid
PFOS: Perfluorooctane sulfonate
PPARα: Peroxisome proliferator-activated receptor alpha
RAG1: Recombination activating gene 1
RAG2: Recombination activating gene 2
RNAseq: RNA sequencing
SEM: Standard error of the mean
SI: Stimulation index
TDAR: T cell-dependent antibody response
TIDAR: T cell-independent antibody response
