# Supplementary material for: Mechanistic insights into PFAS-induced effects on B lymphocyte activation and antibody secretion
Source: Arch Toxicol. 2026 Apr 23;100(7):3073–90. doi: 10.1007/s00204-026-04377-0 (PMC13309518; doi:10.1007/s00204-026-04377-0)
Supplement: Supplementary file 1 — Supplementary file1. [file 204_2026_4377_MOESM1_ESM.pdf]

Supplementary information for:

Mechanistic insights into PFAS-induced effects on B lymphocyte activation and antibody secretion

Martina Iulini[1], Karsten Beekmann[2], Ron L.A.P. Hoogenboom[2], Valentina Galbiati[1], Giulia Russo[3], Francesco Pappalardo[3], Stella Fragki[4], Alicia Paini[4], Emanuela Corsini[1], Aafke W.F. Janssen[2,*]

[1]Laboratory of Toxicology, Department of Pharmacological and Biomolecular Sciences “Rodolfo Paoletti”, Università degli Studi di Milano, Milan, Italy

[2]Wageningen Food Safety Research (WFSR), part of Wageningen University & Research, Wageningen, The Netherlands

[3]Department of Health and Drug Sciences, Università degli Studi di Catania, Italy

[4]esqLABS GmbH, 26683 Saterland, Germany

* **Corresponding Author:**

Aafke W.F. Janssen

Wageningen Food Safety Research (WFSR),

Akkermaalsbos 2

6708 WB Wageningen

The Netherlands

aafke.janssen@wur.nl

Table S1A. The eight most highly upregulated genes in stimulated PBMCs from male donors exposed to PFOA

**Gene Symbol**	**Description**	**Fold Change**	***p* value**	**Ensembl Gene ID**
ELANE	Elastase, neutrophil expressed	5.17	1.22E-03	ENSG00000197561
AREG	Amphiregulin	4.57	3.99E-02	ENSG00000109321
P2RY6	Pyrimidinergic receptor P2Y6	4.52	3.48E-02	ENSG00000171631
NNMT	Nicotinamide N-methyltransferase	4.31	4.85E-02	ENSG00000166741
SBK3	SH3 domain binding kinase family member 3	4.04	3.15E-02	ENSG00000231274
IGLV1-36	Immunoglobulin lambda variable 1-36	3.50	1.02E-02	ENSG00000211655
RGL1	Ral guanine nucleotide dissociation stimulator like 1	3.44	4.41E-02	ENSG00000143344
EMP2	Epithelial membrane protein 2	3.03	3.28E-02	ENSG00000213853

Table S1B. The eight most highly upregulated genes in stimulated PBMCs from male donors exposed to PFOS

**Gene Symbol**	**Description**	**Fold Change**	***p* value**	**Ensembl Gene ID**
CD163	CD163 molecule	48.39	1.56E-02	ENSG00000177575
OLFML2B	Olfactomedin like 2B	20.09	3.38E-02	ENSG00000162745
CXCL12	C-X-C motif chemokine ligand 12	18.47	1.80E-02	ENSG00000107562
C1QC	Complement C1q C chain	14.97	4.93E-02	ENSG00000159189
STAB1	Stabilin 1	13.03	2.89E-02	ENSG00000010327
CXCL9	C-X-C motif chemokine ligand 9	12.09	1.16E-02	ENSG00000138755
VSIG4	V-set and immunoglobulin domain containing 4	10.51	1.67E-02	ENSG00000155659
CDO1	Cysteine dioxygenase type 1	10.35	3.58E-02	ENSG00000129596

Table S1C. The eight most highly upregulated genes in stimulated PBMCs from female donors exposed to PFOA

**Gene Symbol**	**Description**	**Fold Change**	***p* value**	**Ensembl Gene ID**
CD163	CD163 molecule	27.85	1.92E-02	ENSG00000177575
LILRB5	Leukocyte immunoglobulin like receptor B5	12.63	2.45E-02	ENSG00000105609
FCN1	Ficolin 1	10.25	2.89E-02	ENSG00000085265
C1QC	Complement C1q C chain	9.52	3.64E-02	ENSG00000159189
CLEC10A	C-type lectin domain containing 10A	8.50	2.62E-02	ENSG00000132514
SRPX	Sushi repeat containing protein X-linked	8.19	2.73E-02	ENSG00000101955
C1QB	Complement C1q B chain	7.86	3.02E-02	ENSG00000173369
AREG	Amphiregulin	7.63	4.53E-07	ENSG00000109321

Table S1D. The eight most highly upregulated genes in stimulated PBMCs from female donors exposed to PFOS

**Gene Symbol**	**Description**	**Fold Change**	***p* value**	**Ensembl Gene ID**
CD163	CD163 molecule	60.41	4.50E-03	ENSG00000177575
LILRB5	Leukocyte immunoglobulin like receptor B5	17.59	1.07E-02	ENSG00000105609
C1QC	Complement C1q C chain	13.26	1.57E-02	ENSG00000159189
FCN1	Ficolin 1	12.89	1.58E-02	ENSG00000085265
AREG	Amphiregulin	12.66	1.18E-08	ENSG00000109321
CD163L1	CD163 molecule like 1	11.82	1.93E-02	ENSG00000177675
HS3ST2	Heparan sulfate-glucosamine 3-sulfotransferase 2	11.67	8.91E-03	ENSG00000122254
CLEC10A	C-type lectin domain containing 10A	11.02	1.24E-02	ENSG00000132514

Table S2A. The eight most highly downregulated genes in stimulated PBMCs from male donors exposed to PFOA

**Gene Symbol**	**Description**	**Fold Change**	***p* value**	**Ensembl Gene ID**
CST6	Cystatin E/M	-5.24	1.65E-02	ENSG00000175315
DNAJC22	DnaJ heat shock protein family (Hsp40) member C22	-4.94	1.68E-03	ENSG00000178401
PCARE	Photoreceptor cilium actin regulator	-4.49	1.67E-02	ENSG00000179270
TMPRSS4	Transmembrane serine protease 4	-4.23	4.98E-02	ENSG00000137648
DMBX1	Diencephalon/mesencephalon homeobox 1	-4.08	1.24E-02	ENSG00000197587
ADORA1	Adenosine A1 receptor	-3.60	1.39E-02	ENSG00000163485
TMEM92	Transmembrane protein 92	-3.56	4.83E-03	ENSG00000167105
LINC02009	Long intergenic non-protein coding RNA 2009	-3.46	3.87E-02	ENSG00000283646

Table S2B. The eight most highly downregulated genes in stimulated PBMCs from male donors exposed to PFOS

**Gene Symbol**	**Description**	**Fold Change**	***p* value**	**Ensembl Gene ID**
CDH16	Cadherin 16	-12.62	1.45E-03	ENSG00000166589
DMBX1	Diencephalon/mesencephalon homeobox 1	-11.17	1.32E-03	ENSG00000197587
DNAJC22	DnaJ heat shock protein family (Hsp40) member C22	-10.04	2.57E-04	ENSG00000178401
TMPRSS4	Transmembrane serine protease 4	-9.79	1.44E-02	ENSG00000137648
PRSS8	Serine protease 8	-9.73	2.06E-02	ENSG00000052344
ENSG00000286062	Uncategorized gene	-9.33	5.67E-04	ENSG00000286062
CST6	Cystatin E/M	-9.24	5.39E-03	ENSG00000175315
PCARE	Photoreceptor cilium actin regulator	-9.09	4.29E-03	ENSG00000179270

Table S2C. The eight most highly downregulated genes in stimulated PBMCs from female donors exposed to PFOA

**Gene Symbol**	**Description**	**Fold Change**	***p* value**	**Ensembl Gene ID**
F2RL2	Coagulation factor II thrombin receptor like 2	-8.40	7.76E-03	ENSG00000164220
TRIM31	Tripartite motif containing 31	-3.45	3.88E-02	ENSG00000204616
ALDH1A2	Aldehyde dehydrogenase 1 family member A2	-3.36	2.68E-02	ENSG00000128918
MIR3153	MicroRNA 3153	-3.31	1.17E-02	ENSG00000265112
VPS11-DT	VPS11 divergent transcript	-3.20	1.46E-02	ENSG00000272186
ENSG00000287989	Uncategorized gene	-3.04	2.22E-03	ENSG00000287989
LINC01281	Long intergenic non-protein coding RNA 1281	-3.00	3.54E-02	ENSG00000235304
MIR762	MicroRNA 762	-2.45	8.21E-03	ENSG00000211591

Table S2D. The eight most highly downregulated genes in stimulated PBMCs from female donors exposed to PFOS

**Gene Symbol**	**Description**	**Fold Change**	***p* value**	**Ensembl Gene ID**
ALDH1A2	Aldehyde dehydrogenase 1 family member A2	-4.72	1.08E-02	ENSG00000128918
ENSG00000286281	Uncategorized gene	-4.30	3.20E-03	ENSG00000286281
F2RL2	Coagulation factor II thrombin receptor like 2	-4.07	3.29E-02	ENSG00000164220
TGM5	Transglutaminase 5	-4.07	4.08E-02	ENSG00000104055
BCAN	Brevican	-3.76	3.10E-02	ENSG00000132692
ENPP3	Ectonucleotide pyrophosphatase/phosphodiesterase 3	-3.49	5.79E-04	ENSG00000154269
ENSG00000265100	Uncategorized gene	-3.23	1.16E-02	ENSG00000265100
CGREF1	Cell growth regulator with EF-hand domain 1	-3.13	3.51E-02	ENSG00000138028

Table S3A. The eight most significantly regulated genes in PBMCs from male donors exposed to PFOA

**Gene Symbol**	**Description**	***p* value**	**Fold Change**	**Ensembl Gene ID**
TSC22D3	TSC22 domain family member 3	5.51E-06	1.38	ENSG00000157514
CFAP69	Cilia and flagella associated protein 69	4.14E-05	3.58	ENSG00000105792
ENSG00000289198	Uncategorized gene	6.93E-05	-2.79	ENSG00000289198
ENSG00000264007	Uncategorized gene	7.53E-05	9.19	ENSG00000264007
CXCL6	C-X-C Motif chemokine ligand 6	1.32E-04	-6.39	ENSG00000124875
CCL7	C-C Motig chemokine ligand 7	2.03E-04	-5.26	ENSG00000108688
CYP27B1	Cytchrome P450 Family 27 Subfamily B Member 1	5.57E-04	-1.97	ENSG00000111012
MATK	Megakaryocyte-associated tyrosine kinase	6.82E-04	-1.31	ENSG00000007264

Table S3B. The eight most significantly regulated genes in PBMCs from male donors exposed to PFOS

**Gene Symbol**	**Description**	***p* value**	**Fold Change**	**Ensembl Gene ID**
TSC22D3	TSC22 domain family member 3	1.55E-13	2.70	ENSG00000157514
PER1	Period circadian regulator 1	8.41E-12	2.18	ENSG00000179094
FKBP5	FKBP prolyl isomerase 5	1.68E-10	2.81	ENSG00000096060
SMAP2	Small ArfGAP2	1.84E-10	1.82	ENSG00000084070
MATK	Megakaryocyte-associated tyrosine kinase	4.67E-10	-2.25	ENSG00000007264
EMP1	Epithelial membrane protein 1	1.08E-09	-5.08	ENSG00000134531
FLT3	Fms related receptor tyrosine kinase 3	3.84E-09	5.68	ENSG00000122025
YPEL2	Yippee like 2	5.78E-09	2.00	ENSG00000175155

Table S3C. The eight most significantly regulated genes in PBMCs from female donors exposed to PFOA

**Gene Symbol**	**Description**	***p* value**	**Fold Change**	**Ensembl Gene ID**
HSPA1B	Heat shock protein family A Member 1B	4.38E-05	4.02	ENSG00000204388
HSPA6	Heat shock protein family A Member 6	1.36E-04	3.30	ENSG00000173110
HSPA1A	Heat shock protein family A Member 1A	4.56E-04	2.27	ENSG00000204389
CCNB1	Cyclin B1	7.10E-04	1.75	ENSG00000134057
H2AC15	H2A Clustered Histone 15	1.64E-03	2.63	ENSG00000275221
SLC16A14	Solute Carrier Family 16 Member 14	2.18E-03	3.07	ENSG00000163053
HSPH1	Heat shock protein family H Member 1	2.20E-03	1.56	ENSG00000120694
RPL29P14	Ribosomal protein L29 pseudogene 14	2.59E-03	4.98	ENSG00000241112

Table S3D. The eight most significantly regulated genes in PBMCs from female donors exposed to PFOS

**Gene Symbol**	**Description**	***p* value**	**Fold Change**	**Ensembl Gene ID**
FKBP5	FKBP prolyl isomerase 5	2.33E-13	3.30	ENSG00000096060
SMAP2	Small ArfGAP2	3.08E-09	1.88	ENSG00000084070
COL9A2	Collagen Type IX alpha 2 chain	4.59E-08	2.30	ENSG00000049089
KLF9	KLF transcription factor 9	3.77E-07	1.71	ENSG00000119138
TSC22D3	TSC22 domain family member 3	1.29E-06	2.22	ENSG00000157514
RAG1	Recombinant activating gene 1	1.58E-06	-2.54	ENSG00000166349
TXNIP	Thioredoxin interacting protein	2.22E-06	2.31	ENSG00000265972
WAKMAR2	Wound and keratinocyte migration associated LncRNA2	3.61E-06	1.80	ENSG00000237499

Table S4A. The eight most highly upregulated genes in PBMCs from male donors exposed to PFOA

Gene Symbol	Description	Fold Change	*p* value	Ensembl Gene ID
ENSG00000264007	Uncategorized gene	9.19	7.53E-05	ENSG00000264007
ENSG00000235834	Uncategorized gene	4.87	1.24E-02	ENSG00000235834
H4C5	H4 clustered histone 5	4.50	2.97E-03	ENSG00000276966
H2BC3	H2B clustered histone 3	4.47	4.50E-02	ENSG00000276410
RPL29P14	Ribosomal protein L29 pseudogene 14	3.89	3.26E-03	ENSG00000241112
CFAP69	Uncategorized gene	3.58	4.14E-05	ENSG00000105792
ENSG00000279613	Uncategorized gene	3.31	2.51E-03	ENSG00000279613
CLASP1-AS1	CLASP1 Antisense RNA 1	3.23	5.69E-03	ENSG00000265451

Table S4B. The eight most highly upregulated genes in PBMCs from male donors exposed to PFOS

Gene Symbol	Description	Fold Change	*p* value	Ensembl Gene ID
ADORA3	Adenosine A3 receptor	27.31	3.49E-07	ENSG00000282608
OLAH	Oleoyl-ACP-Hydrolase	23.35	6.61E-07	ENSG00000152463
TMIGD3	Transmembrane And Immunoglobulin Domain Containing 3	15.76	5.60E-06	ENSG00000121933
RNASE1	Ribonuclease A family member 1, Pancreatic	12.24	2.90E-03	ENSG00000129538
GPR34	G protein-coupled receptor 34	11.74	6.30E-03	ENSG00000171659
EMP2	Epithelial membrane protein 2	10.29	1.26E-03	ENSG00000213853
LINC02414	Long intergenic non-protein coding RNA 2414	9.36	1.62E-03	ENSG00000256424
ENSG00000256209	Uncategorized gene	8.81	3.31E-03	ENSG00000256209

Table S4C. The eight most highly upregulated genes in PBMCs from female donors exposed to PFOA

Gene Symbol	Description	Fold Change	*p* value	Ensembl Gene ID
H4C5	H4 Clustered Histone 5	5.24	3.21E-03	ENSG00000276966
RPL29P14	Ribosomal protein L29 pseudogene 14	4.98	2.59E-03	ENSG00000241112
NCF1B	Neurophil cytosolic factro 1B	4.72	2.62E-02	ENSG00000182487
CNTD1	Cyclin N-terminal domain containing 1	4.25	3.02E-03	ENSG00000176563
ENSG00000264701	Uncategorized gene	4.17	3.48E-02	ENSG00000264701
HSPA1B	Heat shock protein family A Member 1B	4.02	4.38E-05	ENSG00000204388
RMDN2-AS1	RMDN2 Antisense RNA 1	3.33	2.81E-02	ENSG00000235848
HSPA6	Heat shock protein family A Member 6	3.30	1.36E-04	ENSG00000173110

Table S4D. The eight most highly upregulated genes in PBMCs from female donors exposed to PFOS

Gene Symbol	Description	Fold Change	*p* value	Ensembl Gene ID
ADORA3	Adenosine A3 receptor	103.53	6.31E-04	ENSG00000282608
FFAR4	Free fatty acid receptor 4	40.00	2.76E-02	ENSG00000186188
TMIGD3	Transmembrane and immunoglubuline domain containing 3	19.12	1.19E-04	ENSG00000121933
SRPX	Sushi repeat containing protein X-linked	18.44	5.89E-03	ENSG00000101955
RPL7AP64	Ribosomal protein L7a pseudogene 64	18.26	9.96E-03	ENSG00000213876
CDO1	Cysteine dioxygenase type 1	17.16	2.71E-02	ENSG00000129596
OLFML3	Olfactomedin like 3	16.67	6.67E-02	ENSG00000116774
GPR34	G protein-coupled receptor 34	14.82	5.40E-02	ENSG00000171659

Table S5A. The eight most highly downregulated genes in PBMCs from male donors exposed to PFOA

**Gene Symbol**	**Description**	**Fold Change**	***p* value**	**Ensembl Gene ID**
IL6	Interleukin 6	-9.19	1.83E-03	ENSG00000136244
CXCL6	C-X-C Motif chemokine ligand 6	-6.39	1.32E-04	ENSG00000124875
CCL7	C-C Motif chemokine ligand 7	-5.26	2.03E-04	ENSG00000108688
CXCL1	C-X-C Motif chemokine ligand 1	-4.43	1.92E-02	ENSG00000163739
CCL19	C-C Motif chemokine ligand 19	-3.61	3.34E-02	ENSG00000172724
ENSG00000287037	Uncategorized gene	-3.38	1.41E-02	ENSG00000287037
ENSG00000289390	Uncategorized gene	-3.18	1.86E-02	ENSG00000289390
ENSG00000228403	Uncategorized gene	-3.11	3.90E-02	ENSG00000228403

Table S5B. The eight most highly downregulated genes in PBMCs from male donors exposed to PFOS

**Gene Symbol**	**Description**	**Fold Change**	***p* value**	**Ensembl Gene ID**
CXCL5	C-X-C Motif chemokine ligand 5	-38.93	1.52E-04	ENSG00000163735
CXCL6	C-X-C Motif chemokine ligand 6	-37.49	5.25E-06	ENSG00000124875
SLC12A5-AS1	SLC12A5 and MMP9 antisense RNA 1	-29.33	2.33E-08	ENSG00000204044
MMP9	Matrix metallopeptidase 12	-27.84	1.04E-06	ENSG00000100985
OCSTAMP	Osteoclast stimulatory transmembrane protein	-19.55	3.35E-05	ENSG00000149635
CXCL1	C-X-C Motif chemokine ligand 1	-19.39	1.57E-03	ENSG00000163739
CCL7	C-C Motig chemokine ligand 7	-16.98	6.92E-06	ENSG00000108688
CHI3L1	Cristinase 3	-16.46	1.24E-07	ENSG00000133048

Table S5C. The eight most highly downregulated genes in PBMCs from female donors exposed to PFOA

**Gene Symbol**	**Description**	**Fold Change**	***p* value**	**Ensembl Gene ID**
VIT	Vitrin	-5.11	3.24E-02	ENSG00000205221
IGHV4-4	Immunoglobulin heavy variable 4-4	-5.03	2.16E-02	ENSG00000276775
TDRD1	Tudor domain containing 1	-3.98	3.67E-02	ENSG00000095627
ERFE	Erythroferrone	-3.96	9.39E-03	ENSG00000178752
ENSG00000269583	Uncategorized gene	-3.59	2.37E-02	ENSG00000269583
IGFN1	Immunoglobulin like and fibronectn type III domain containing 1	-3.55	2.71E-02	ENSG00000163395
IGLV10-54	Immunoglobulin lambda variable 10-54	-3.53	1.85E-02	ENSG00000211642
RPL35AP20	Ribosomal protein L35a pseudogene 20	-3.39	1.97E-02	ENSG00000232946

Table S5D. The eight most highly downregulated genes in PBMCs from female donors exposed to PFOS

**Gene Symbol**	**Description**	**Fold Change**	***p* value**	**Ensembl Gene ID**
CXCL6	C-X-C Motif chemokine ligand 6	-22.86	4.25E-02	ENSG00000124875
MMP12	Matrix metallopeptidase 12	-12.49	4.20E-02	ENSG00000262406
NDP	Norrin cystine knot growth factor NDP	-12.01	5.76E-03	ENSG00000124479
ENSG00000287561	Uncategorized gene	-11.27	2.08E-02	ENSG00000287561
OCSTAMP	Osteoclast stimulatory transmembrane protein	-11.00	2.35E-03	ENSG00000149635
IGHV4-4	Immunoglobulin heavy variable 4-4	-10.89	5.48E-03	ENSG00000276775
IGFN1	Immunoglobulin like and fibronectn type III domain containing 1	-10.31	2.26E-03	ENSG00000163395
INHBA	Inhibin subunit beta A	-10.30	2.27E-03	ENSG00000122641

Table S6. Overview of z-score of suggested upstream regulators (p-value of overlap <0.05) related to NFκB in PBMCs exposed to 12.6 6 μg/mL PFOA or PFOS, and 150 μg/mL DEX.

Sex	Exposure	Upstream Regulator	Activation z-score	p-value of overlap
Male	DEX	NFkB (complex)	-4.73	2,02E-19
		NFkB (family)	-2.75	1,77E-05
		NFKB1	-3.41	2,39E-16
		NfkB1-RelA	-2.61	1,02E-03
		NfkB-RelA	-2.76	6,54E-04
	PFOA	NFkB (complex)	-2.20	2,71E-07
		NFKB1	-2.77	2,15E-07
	PFOS	NFkB (complex)	-6.70	1,69E-25
		NFkB (family)	-3.48	4,52E-05
		NFKB1	-3.77	5,42E-19
		NfkB1-RelA	-3.64	5,23E-08
		NFKB2	-2.23	4,28E-07
		NfkB-RelA	-2.98	1,51E-05
Female	PFOS	NFkB (complex)	-3.81	3,84E-07
		NFkB (family)	-3.08	8,04E-03
		NFKB1	-3.81	7,12E-06
		NfkB-RelA	-2.43	2,61E-02

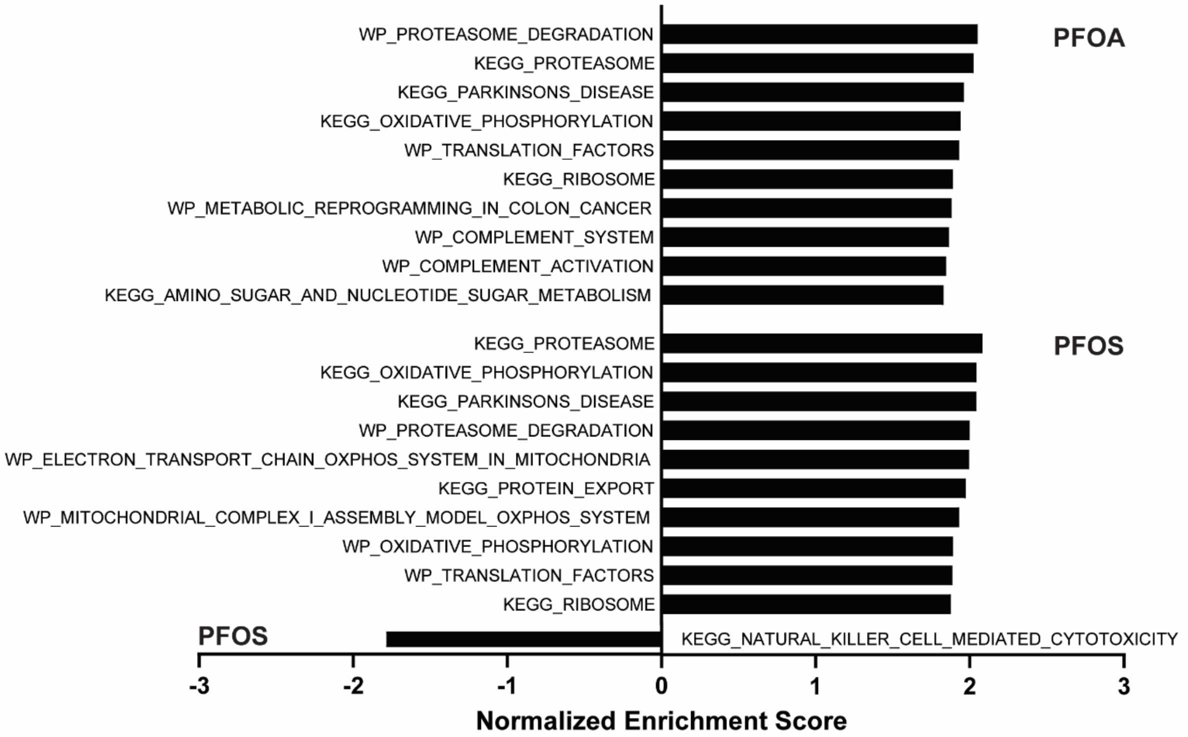

Supplementary Figure 1. Top 10 gene sets induced or repressed in activated PBMCs from male donors after 7-day exposure to PFOA or PFOS. The top 10 gene sets were determined through gene set enrichment analysis and based on their normalized enrichment scores.

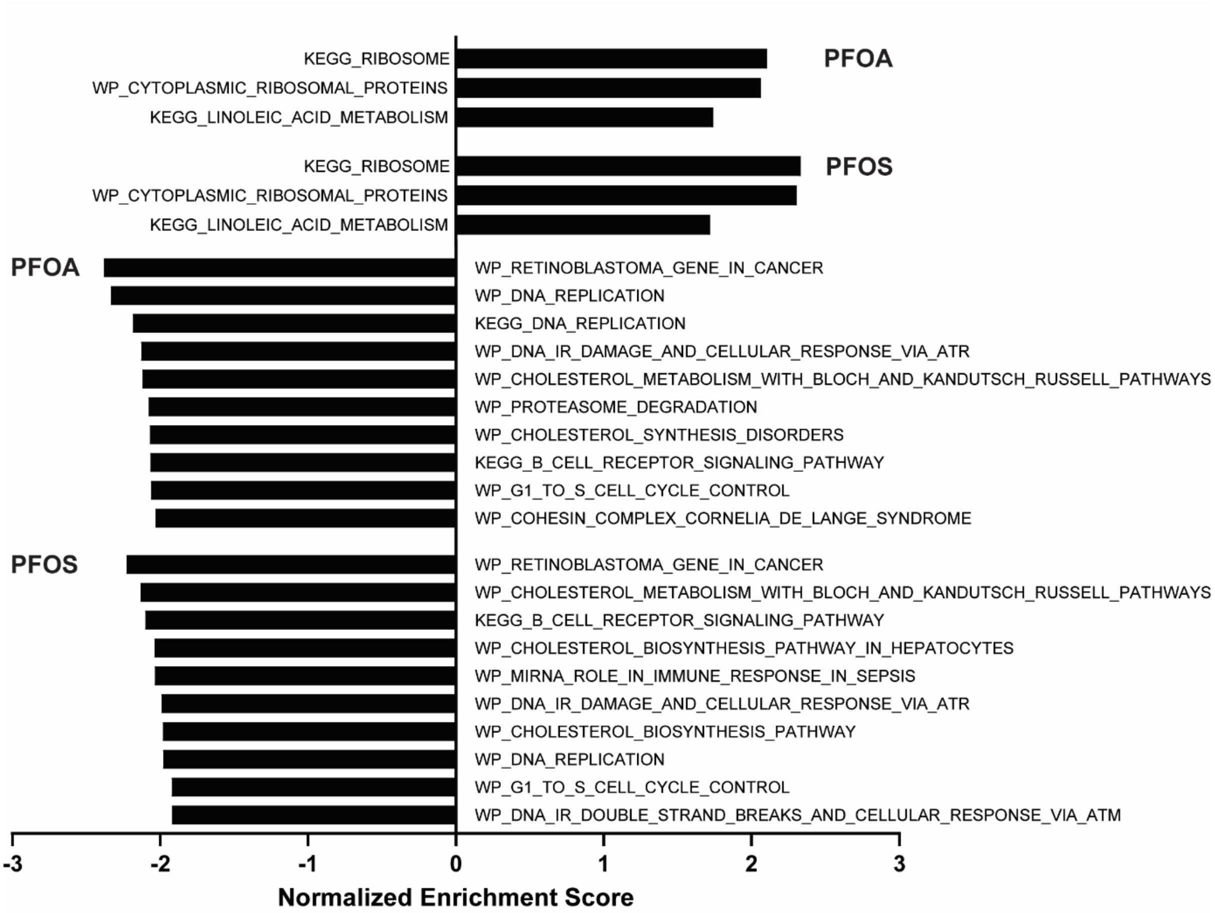

Supplementary Figure 2. Top 10 gene sets induced or repressed in activated PBMCs from female donors after 7-day exposure to PFOA or PFOS. The top 10 gene sets were determined through gene set enrichment analysis and based on their normalized enrichment scores.
